# ST-Elevation Myocardial Infarction With Occluded Culprit Coronary Artery in a Young Patient Recovered From Mild COVID-19: A Case Report

**DOI:** 10.7759/cureus.29258

**Published:** 2022-09-17

**Authors:** Juan Manuel Muñoz Moreno, Anthony Ramos-Yataco, Erik Villanueva Garcia, Carlos Holguin Palacios, Gina Sánchez Sánchez

**Affiliations:** 1 Cardiology, Hospital Nacional Edgardo Rebagliati Martins, Lima, PER; 2 Internal Medicine, Hospital Ricardo Cruzado Rivarola, Nasca, PER; 3 Cardiology, Instituto Nacional Cardiovascular, Lima, PER

**Keywords:** thrombosis, st elevation myocardial infarction, percutaneous coronary intervention, drug-eluting stents, covid-19

## Abstract

ST-elevation myocardial infarction (STEMI) is a cardiovascular emergency that requires an early reperfusion strategy to reduce mortality and hemodynamic, mechanical, and electrical complications. STEMI is more frequent in men older than 40 years with well-known cardiovascular risk factors such as hypertension, diabetes mellitus, dyslipidemia, and smoking. The coronavirus disease 2019 (COVID-19) changed this reality worldwide due to the fact that STEMI cases associated with severe forms of COVID-19 began to be reported, which generally affected the older adult population; in contrast, there is still limited data on young healthy patients recovering from mild COVID-19. The exact mechanism behind the association remains unclear. We present a case of a healthy 29-year-old man with a history of mild COVID-19, diagnosed by reverse-transcription polymerase chain reaction 20 days before his admission with inferior STEMI. Coronary angiography revealed an occluded mid-right coronary artery, and he was successfully treated with a drug-eluting stent. The patient evolved favorably and was discharged on the fifth day of hospitalization.

## Introduction

The coronavirus disease 2019 (COVID-19) pandemic caused by the severe acute respiratory syndrome coronavirus-2 (SARS-CoV-2) has caused about 6.5 million deaths worldwide [1]. Currently, the Peruvian Ministry of Health reported around 4.1 million cases resulting in 215,639 deaths, representing a case-fatality rate of 5.3%, which is the highest in the world [1,2]. COVID-19 causes multiple cardiovascular complications, such as myocarditis, atrioventricular blocks, arrhythmias, sudden cardiac death, and myocardial infarction [3]. 

ST-elevation myocardial infarction (STEMI) related to COVID-19 is mainly associated with its severe form, and in some cases, a culprit lesion could not be identified in those patients who underwent coronary angiography [3]. Conversely, there are only limited experiences in young healthy adults who recovered from mild COVID-19. In this case report, we present a case of STEMI in a young healthy male who recovered from mild COVID-19, underwent coronary angiography, and found an occluded culprit coronary artery, which was successfully treated, achieving a favorable outcome.

## Case presentation

**Figure 1:**
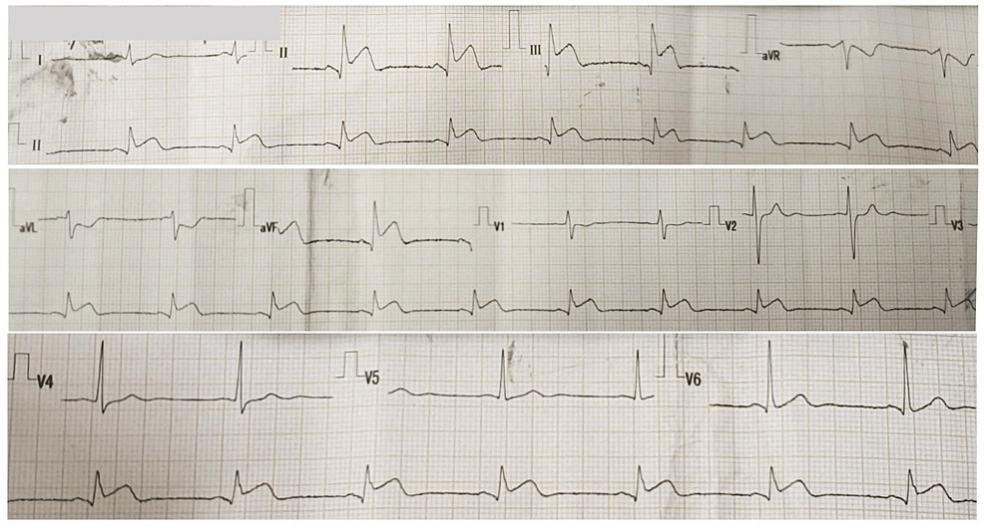
Initial electrocardiogram. Sinus rhythm (56 beats/min), ST-segment elevation in inferior leads (II, III, aVF), and ST-segment depression in reciprocal leads (I, aVL).

A 29-year-old man presented to our hospital reporting 13 hours of severe chest tightness. His past medical history was positive for mild COVID-19, diagnosed by reverse-transcription polymerase chain reaction 20 days before admission. Vital signs at admission and physical examination were unremarkable. An electrocardiogram (ECG) showed sinus rhythm and ST-segment elevation in inferior leads (II, III, and aVF) (Figure 1). Serum immunochromatography test for SARS-CoV-2 was positive for IgG, troponin level was elevated (TnT 1.92 ng/ml; normal <0.1 ng/ml). 

In the setting of a patient with typical chest pain, ECG changes consistent with acute ischemia, and elevated cardiac enzymes, the diagnosis of inferior STEMI was established. He received a loading dose of aspirin 300 mg, clopidogrel 600 mg, and unfractionated heparin (UFH) 4000 IU IV. Furthermore, in a healthy young male, without traditional cardiovascular risk factors and with a history of recovered mild COVID-19, the possibility of COVID-19-associated STEMI was raised.

**Figure 2:**
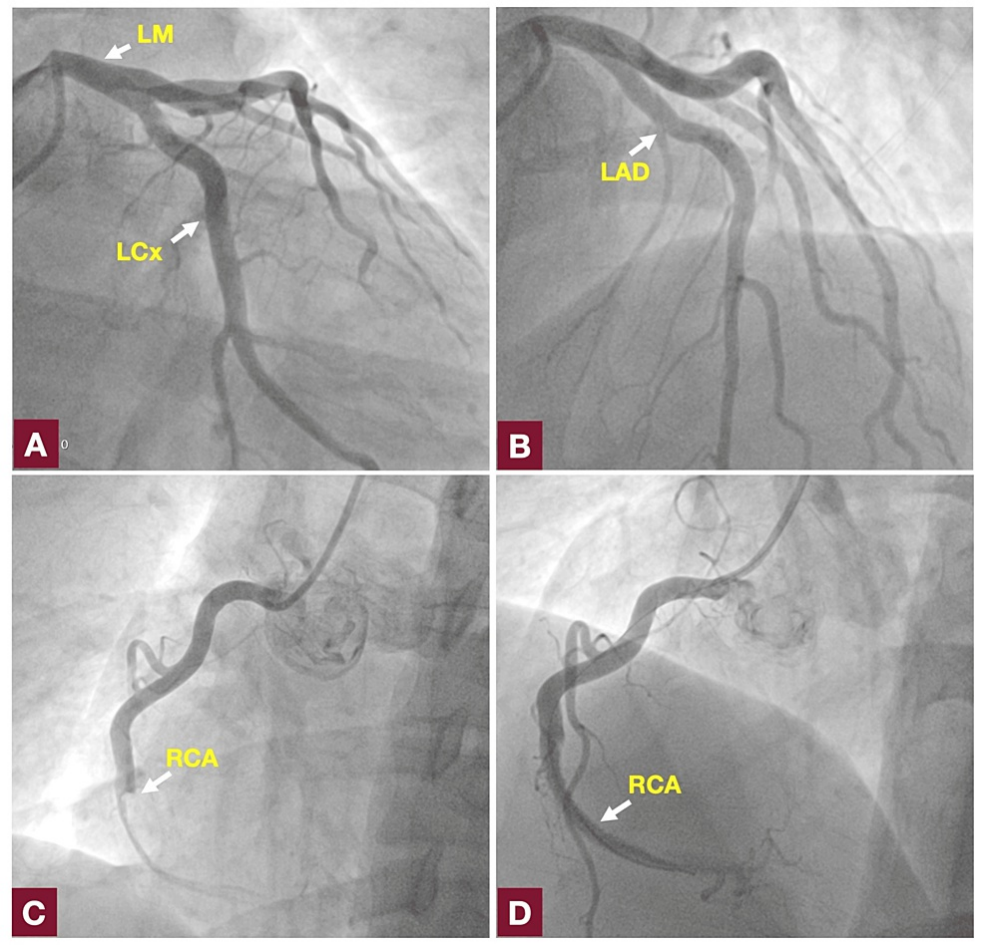
Cardiac catheterization. (A) and (B) Left main coronary artery (LM), left circumflex coronary artery (LCx), and left anterior descending artery (LAD) without lesions. (C) Occluded the mid-right coronary artery (RCA). (D) Successful coronary post-angioplasty, with a significant thrombotic burden toward the distal segment of the RCA.

A chest x-ray was normal. Transthoracic echocardiogram (TTE) showed a left ventricular ejection fraction (LVEF) of 50%, right ventricular fractional area change (RVFAC) of 40%, with hypokinesia of the basal inferior, basal inferolateral, and mid-inferior segments. The patient was referred to the cath lab for primary percutaneous coronary intervention (PCI). Coronary angiography revealed total occlusion of the mid-right coronary artery (RCA) without lesions in other arteries (Figures 2A, 2B, 2C). He was treated with a drug-eluting stent, thrombolytic in myocardial infarction (TIMI) 3 flow was restored, and a significant thrombotic burden was visualized at the distal artery (Figure 2D). Therefore, since glycoprotein IIb/IIIa inhibitors were not available in our country, therapeutic anticoagulation was started with UFH infusion for 48 hours, adjustable according to activated thromboplastin time from 50 to 70.

**Figure 3:**
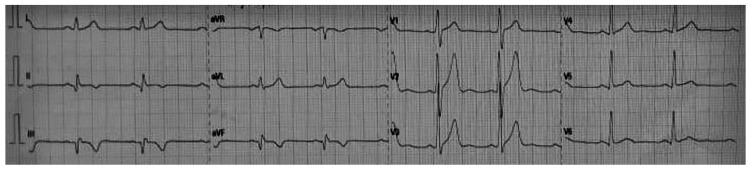
Pre-discharge electrocardiogram. Sinus rhythm (68 beats/min), negative T waves in inferior leads, Q waves in III-aVF, and peaked T waves in V2-V3.

His clinical status improved; he was asymptomatic post-procedure and received dual antiplatelet therapy (DAPT), a statin, and beta-blockers (BBs). Furthermore, secondary causes of STEMI were ruled out, such as dyslipidemia, diabetes mellitus, cocaine intake, and thrombophilia (protein C, protein S, antithrombin III, factor V Leiden, and autoantibodies were all within the normal range). Before discharge, the ECG showed negative T waves in the inferior leads and Q waves in the III and aVF (Figure 3).

The evolution of the patient was favorable. TTE before discharge showed improvement in LVEF of up to 60%, with preserved lower segment motility. He was discharged on the fifth day of hospitalization with DAPT, statin, and BB. Currently, after six months of outpatient follow-up, he remains asymptomatic on his medication regimen.

## Discussion

COVID-19 causes multiple cardiovascular complications, including STEMI. Throughout the COVID-19 pandemic, STEMI cases associated mainly with severe forms of COVID-19, generally affecting the older adult population, were reported, and in some cases, a culprit lesion could not be identified on angiography coronary [3]. Nevertheless, there is still limited data on healthy young patients who have recovered from mild COVID-19. We present the case of a young patient recovering from mild COVID-19 who developed STEMI with an apparent occluded culprit vessel.

The association described between COVID-19 and STEMI was published in multiple case reports, including most patients with different comorbidities such as diabetes and hypertension, who also had a mean age of 58.3 years, and usually presented with severe clinical symptoms of COVID-19 [4]. A case series reported by Bangalore et al. identified 18 patients with COVID-19-related STEMI. The median age was 63 years, and out of those, only 67% had obstructive coronary disease [5].

It is believed that several mechanisms are involved in STEMI among patients with COVID-19. It predisposes to a hypercoagulable state through endothelial damage and enhanced immune response through cytokine storm, which affects cardiomyocytes and triggers the coagulation cascade. Furthermore, SARS-CoV-2 binds to angiotensin-converting enzyme-2 receptors found in cardiac cells, which may cause direct cardiomyocyte damage [6,7]. Our patient did not have evidence of atherosclerotic plaque in coronary angiography; thus, we hypothesize that a persistent hypercoagulable and proinflammatory state related to prior mild COVID-19 may have predisposed the patient to STEMI.

During this COVID-19 pandemic, both the American College of Cardiology (ACC) and the European Society of Cardiology (ESC) recommends primary PCI as the standard of care for patients with STEMI when it can be provided in a timely manner [8,9]. The maximum delay from STEMI diagnosis to reperfusion should continue to be 120 min, according to the ESC [9]. When the target time cannot be met and it is not contraindicated, fibrinolysis should be performed in those patients with less than 12 hours of STEMI evolution, highlighting that the greatest benefit occurs in the first two hours of symptom onset [9,10]. Likewise, they recommend performing a TTE before cardiac catheterization to determine alterations in the contractile wall only when a STEMI diagnosis is not clear [8,9]. In accordance with the recommendations above, TTE was performed on our patient, observing alterations in contractile motility in the inferior wall. Primary PCI was performed since he came to our center with 13 hours of evolution. This delay can be explained because, in our country, the availability of specialized PCI centers is limited.

There is a high thrombotic burden in patients suffering from COVID-19, so higher doses of heparin are required to achieve therapeutic anticoagulation [11]. Our patient received two more days of UFH post primary PCI based on our hospital protocol.

## Conclusions

Mild COVID-19 can cause STEMI in young patients without traditional cardiovascular risk factors. STEMI, according to the known pathophysiology of acute coronary syndrome, can manifest with an occluded culprit coronary artery, even without the presence of a previous atherosclerotic plaque, as occurs in COVID-19. A hypercoagulable and proinflammatory state through endothelial damage and cytokine storm, respectively, have been hypothesized for this association. However, the exact mechanism is not yet fully understood.

Therefore, clinicians should be aware that STEMI with an occluded culprit coronary artery may occur in healthy young patients who have recovered from mild COVID-19 and should perform early coronary angiography in order to reduce mortality and hemodynamics, mechanical, and electrical complications.
